# Multidisciplinary Care for Gender-Diverse Youth: A Narrative Review and Unique Model of Gender-Affirming Care

**DOI:** 10.1089/trgh.2016.0009

**Published:** 2016-07-01

**Authors:** Diane Chen, Marco A. Hidalgo, Scott Leibowitz, Jennifer Leininger, Lisa Simons, Courtney Finlayson, Robert Garofalo

**Affiliations:** ^1^ Division of Adolescent Medicine, Ann & Robert H. Lurie Children's Hospital of Chicago, Chicago, Illinois.; ^2^ Department of Child and Adolescent Psychiatry, Ann & Robert H. Lurie Children's Hospital of Chicago, Chicago, Illinois.; ^3^ Department of Psychiatry and Behavioral Sciences, Northwestern University Feinberg School of Medicine, Chicago, Illinois.; ^4^ Department of Pediatrics, Northwestern University Feinberg School of Medicine, Chicago, Illinois.; ^5^ Division of Endocrinology, Ann & Robert H. Lurie Children's Hospital of Chicago, Chicago, Illinois.

**Keywords:** gender affirmative care, gender diversity, gender dysphoria, multidisciplinary care, transgender

## Abstract

Heightened public awareness about gender diversity in childhood and adolescence has resulted in more youth and families seeking medical and mental health services. In response to these needs, there has been nationwide growth in specialized multidisciplinary clinics treating gender-diverse and transgender youth. Despite general agreement that comprehensive treatment is best delivered through a multidisciplinary team by both medical and mental health clinicians with gender-related expertise and familiarity with child and adolescent development, there is currently no consensus regarding the best approach to clinical care with gender-diverse and transgender youth. In this article, we provide a narrative review of the gender affirmative model guiding our clinical practice and describe the development of our unique model of affirming care within the Gender and Sex Development Program at the Ann & Robert H. Lurie Children's Hospital of Chicago.

## Introduction

The first multidisciplinary pediatric gender clinic in the United States was formally established in 2007.^1^ Since that time, heightened public awareness about gender diversity has resulted in more youth and families seeking medical and mental health services to better understand youths' gender development and identities, social gender transition, and/or physical transition to an affirmed gender.^2–4^ In response to these needs, there has been nationwide growth in specialized clinics for gender-diverse and transgender youth.^5^ However, despite general agreement that comprehensive care is best delivered through a multidisciplinary team that comprises both medical and mental health clinicians,^6–9^ there is currently no consensus regarding the best approach to clinical care with gender-diverse and transgender youth. In this article, we provide a narrative review of the gender affirmative model^10^ guiding our clinical practice and describe the development of our unique model of affirming care within the Gender and Sex Development Program (GSDP) at the Ann & Robert H. Lurie Children's Hospital of Chicago (formerly known as Children's Memorial Hospital).

## Gender-Affirming Care

The terms “gender-diverse” and “transgender” refer to individuals whose gender expressions or identities do not conform to culturally defined norms associated with their birth-assigned sex. Many, but not all, gender-diverse and transgender individuals experience gender dysphoria (GD), which refers to emotional distress stemming from incongruence between an individual's birth-assigned sex and their subjective sense of self as male, female, or an alternate gender.^8^ GD replaced “Gender Identity Disorder” in the *Diagnostic and Statistical Manual of Mental Disorders, version 5* (DSM-5), which shifts the diagnostic focus from nonconforming behavioral expressions or identities to clinically significant distress stemming from gender incongruence.^11^

Consistent with the gender affirmative model,^10^ our clinical practice views variations in both gender identities and behavioral expressions as part of expected human diversity and not inherently pathological in nature. Our affirming philosophy supports gender-diverse youth living as they feel most comfortable and promotes exploration of gender without presuming a fixed trajectory with regard to gender identity. As such, we prioritize a flexible, individualized, and comprehensive approach to ensure gender-diverse youth and their families receive not only medical and mental health treatment when indicated but also adjunctive services that promote overall adjustment and well-being within peer-, school-, and community-based systems.

Medical protocols used by multidisciplinary teams typically follow recommendations by the World Professional Association of Transgender Health (WPATH)^8^ and Endocrine Society.^6^ Before starting puberty, there is no indication to medically treat youth with GD. Some, but not all, prepubertal youth with GD may undergo a social gender transition, which involves reversible changes to an individual's external presentation (e.g., clothing, hairstyle, name/gender pronoun change). Among prepubertal gender-diverse youth without GD, treatment may include psychoeducation and support around developing problem solving and coping skills to counteract the potential effects of stigma that occur in response to the child's gender diversity.

For youth with gender-related body dissatisfaction, medical interventions may be considered once pubertal development has started, with distinct recommendations for early (Tanner stages 2–3) and late (Tanner stages 4–5) pubertal youth.^6,8,12^ Gonadotropin-releasing hormone analogues (GnRHa) represent a reversible intervention that suppresses sex hormone production (i.e., puberty “pausers” or “blockers”), thereby preventing progression of pubertal changes.^6,13^ If used early, GnRHa can alleviate distress related to impending pubertal changes (some of which are irreversible) and, for those who subsequently proceed with gender-affirming hormones, reduce the need for invasive procedures to achieve a desired phenotype. For late pubertal adolescents, gender-affirming hormones may be administered to facilitate development of secondary sex characteristics that are congruent with gender identity.^12,13^ Estrogen, sometimes in conjunction with an androgen-receptor blocker, is used for feminization, and testosterone is used for masculinization. Before initiating these interventions, an evaluation by a licensed mental health provider documenting readiness for treatment is recommended.^6,8^

## Lurie Children's Gender and Sex Development Program Development

GSDP was established in July 2013 through a 3-year start-up gift from a private philanthropic foundation. The multidisciplinary team of pediatric specialists initially included an adolescent medicine physician, an endocrinologist, and two clinical psychologists, as well as a program manager (PM) with a Master's-level background in education. The program supported its psychologists in receiving extensive training and consultation with renowned psychologists from academic gender centers that serve gender-diverse youth from early childhood through young adulthood. Together with published descriptions of pediatric gender clinics in the United States^1,4^ and the Netherlands,^2^ these trainings informed the development of GSDP's core behavioral health services. In October and November 2013, two additional clinicians with transgender health expertise joined the team—an adolescent medicine physician and a child–adolescent psychiatrist, respectively, which allowed for expansion of clinic hours and service offerings. In January 2015, a social worker was hired primarily to aid families in locating community resources in the context of our PM's expanding role in community outreach efforts. Finally, in December 2015, an Advanced Practice Nurse (APN) specializing in adolescent medicine was hired to coordinate clinical research efforts and support physicians in provision of medical care.

Financial support for GSDP consists of clinical revenue, institutional support, and philanthropic funding. All medical services are supported through clinical revenue. Behavioral health services outside of gender development clinic (GDC) are supported through clinical revenue, with psychologists' time in GDC supported through institutional “buy out.” Philanthropic support funds the following: (1) PM's salary, (2) protected research and program development time (10–50%) for each GSDP specialist, thereby increasing our capacity to develop adjunctive programming, and (3) service provision for uninsured or underinsured patients on a case-by-case basis.

## Clinical Practice

### Intake

Our PM is the initial point of contact for all families interested in establishing care with GSDP. The PM conducts the initial telephone intake and gathers background information about parental (or patient, if the patient is of majority age) concerns and clinical service needs, provides information about services offered by GSDP, and connects families to community-based legal, school, and/or supportive resources as indicated. Patients and families can establish clinical care with our program either through multidisciplinary GDC or direct referral to the behavioral health team. Clinical appointments are family initiated. Eligible patients are youth under 25 years of age, and parental participation is required for youth under the age of 18. Youth and families can access adjunctive services without pursuing clinical care with our providers.

### Multidisciplinary GDC

GDC, a twice-monthly half-day clinic, has been the primary avenue through which patients establish clinical care in our program. Providing multidisciplinary services in GDC are our adolescent medicine physicians, APN, psychologists, and endocrinologist. All new patients are seen by a medical staff member (adolescent medicine physician, APN, or endocrinologist) and a psychologist together at their initial appointment, which allows for joint interviewing and limits the need for families to repeat gender and psychosocial histories. This model also facilitates close collaboration among medical and behavioral health staff following a patient's initial clinical visit. Our endocrinologist typically plays a consulting role in GDC, but due to patient volume acts as the primary GSDP physician for a small caseload of primarily early pubertal youth.

All parents and patients ages 12 years and older complete a battery of psychosocial and gender-specific measures (Tables 1–3) at their initial GDC appointment. Together with a clinical interview, these measures provide an overview of a patient's gender development and current understanding of gender identity, body satisfaction, level of support for gender diversity, and emotional and behavioral functioning. This initial appointment, lasting ∼1.5 h, including time to complete measures, does not represent a comprehensive diagnostic evaluation or evaluation of readiness for medical or surgical interventions. Instead, the aim of the appointment is to inform treatment planning by identifying the patient's and parents' treatment goals and collecting information about gender development and psychosocial functioning to guide clinical recommendations.

**Table 1.** **Battery of Measures for Patients Ages 11 Years and Younger**

Measures
Child Symptom Inventory-4^18,19,a^
Early Childhood Symptom Inventory-4^20,b^
Gender Identity Questionnaire for Children^21,22^
Parenting Stress Index, Short Form^23^
Parental Support for Gender Variance^c^

All measures are parent-report.

^a^ For ages 6 years and older.

^b^ For ages 3–5 years.

^c^ Forbes C, Banisaba B. Trans Youth Parental Support Measure. 2010. Unpublished manuscript.

**Table 2.** **Battery of Measures for Patients Ages 12–18 Years**

Measure
Youth self-report
Youth Inventory-4^24^
Utrecht Gender Dysphoria Scale, Adolescent Version^25^
Body Image Scale^26^
Transgender Congruence Scale^27^
Gender Minority Stress and Resiliency Scale^28^
Perceptions of Parental Support for Gender Variance^a^
Parent report
Adolescent Symptom Inventory-4^29,30^
Parental Support for Gender Variance^a^

^a^ Forbes C, Banisaba B. Trans Youth Parental Support Measure. 2010. Unpublished manuscript.

**Table 3.** **Battery of Measures for Patients Ages 19 Years and Older**

Measure
Youth self-report
Brief Symptom Inventory-18^31^
Utrecht Gender Dysphoria Scale^25^
Body Image Scale^26^
Transgender Congruence Scale^27^
Gender Minority Stress and Resiliency Scale^28^
Perception of Parental Support for Gender Variance^b^
Parent report^a^
Parental Support for Gender Variance^b^

^a^ Obtained only with patient's consent if parent accompanies the adult patient to visit.

^b^ Forbes C, Banisaba B. Trans Youth Parental Support Measure. 2010. Unpublished manuscript.

Patients and families have various goals for establishing care, with some seeking education on GD course and whether and when to consider a social or medical gender transition. In these cases, the initial GDC visit may include psychoeducation about gender development and gender diversity, description of consensus (or lack thereof) on social gender transition for prepubertal children with GD, and indications for medical intervention. Other patients, upon their initial visit to GDC, are already living in their affirmed gender role and are interested in pursuing medical transition. Care is made to individualize treatment recommendations based on clinical information obtained from the initial visit, which may include recommended follow-up with behavioral health staff (for therapy focused on gender exploration or evaluation of readiness for medical intervention) or medical staff (for psychoeducation regarding hormonal intervention).

For youth and families interested in initiating GnRHa or gender-affirming hormones, the initial visit includes a discussion of GDC protocol and, when indicated, our clinical practice of considering gender-affirming hormones for youth who are cognitively and emotionally prepared around the age of 14. This practice varies from the Endocrine Society recommendation to wait until around age 16 for hormone treatment.^6^ Our rationale is explained—delaying exposure to sex steroids may decrease bone density among those on GnRHa.^14^ In addition, by age 16 most adolescents will have reached pubertal maturity, and delaying a gender-congruent puberty among transgender youth who are emotionally and cognitively mature enough to assent to hormone treatment before age 16 may have negative emotional and social consequences. We describe the steps to be completed during medical follow-up appointments before starting treatment, including (1) a comprehensive medical history, physical examination, and laboratory work, and for those starting GnRHa, a bone age and DEXA scan, (2) psychoeducation regarding anticipated effects, timeline of changes, and risks of hormonal therapy, and (3) written informed consent from the patient and, if the patient is a minor, consent from the legal guardian(s). In minors for whom there is more than one legal guardian, we make an effort to obtain written consent from both guardians. When there is disagreement, our team provides direct support in navigating discussions with the goal of reaching consensus. When agreement is not possible *and* it is determined that the minor will suffer emotionally without intervention, we have accepted written consent from a single guardian. In certain instances, consultation with a hospital ethicist has been helpful.

In addition to medical follow-up, we describe the need for a letter documenting readiness for medical treatment per criteria outlined by WPATH^8^ from a licensed mental health provider with at least masters' level education. GSDP does not require patients to be engaged in therapy for a specific length of time, recognizing that ongoing therapy may not be clinically indicated for all patients presenting for medical transition, nor do we require that readiness assessments be conducted by GSDP behavioral health clinicians, although this option is offered. For patients in care with community-based clinicians, permission is obtained for GSDP behavioral health staff to collaborate on patient care, including ongoing consultation around conducting a readiness assessment. For patients without established community-based clinicians, families may choose to see a GSDP behavioral health clinician or obtain referrals for gender-affirming clinicians in the community. Medical follow-up typically occurs outside of GDC during general outpatient clinic hours; however, patients with psychiatric/psychosocial complexity typically follow up in GDC, where our psychologists are present to address emergent clinical needs.

In the 31 months since implementing this model, we have seen 220 patients in GDC with ∼10–12 new patients seen each month. Fifty-four patients (37 birth-assigned males) were ages 11 and under, 154 patients (59 birth-assigned males) were ages 12–18 years, and 12 patients (4 birth-assigned males) were ages 19 and older at the time of their initial appointment. Thirty-four patients have been started on GnRHa for pubertal suppression, 20 have been started on estrogen, and 62 have been started on testosterone.

### Behavioral health services

As highlighted, the behavioral health team is comprised of two psychologists and a child–adolescent psychiatrist. Services offered by psychologists and the psychiatrist are available to patients up to age 25 and 18 years, respectively. Providers conduct comprehensive diagnostic evaluations typically ranging from 2 to 4 sessions, the results of which may inform ongoing psychotherapy within GSDP. These providers also share responsibilities in conducting services that may be adjunctive to behavioral healthcare patients are receiving elsewhere, including evaluations for GD and assessments of readiness for medical treatment. Referrals for comprehensive evaluations are either (1) recommended following an initial GDC appointment for patients without community-based clinicians who are exhibiting behavioral/emotional difficulties or in need of a readiness assessment for medical treatment or (2) family initiated, including those establishing care with GSDP through behavioral health services versus GDC and patients preferring to augment care with community-based clinicians with gender-specific services with GSDP behavioral health staff.

The program psychiatrist offers supportive psychotherapy with a focus on gender-related issues. The program psychologists also provide supportive therapy in addition to treating co-occurring psychiatric conditions with evidence-based approaches (e.g., cognitive behavioral therapy). In cases when both evidence-based therapy and psychopharmacotherapy are indicated, a psychologist and the psychiatrist may comprise a patient's treatment team—the psychologist serving as a primary therapist and the psychiatrist offering psychotropic medication management. To date, 103 patients have had initial clinical appointments with a GSDP behavioral health clinician outside of GDC. If and when these patients are interested in pursuing hormonal treatment, a referral is made to one of our medical staff to be scheduled outside of GDC.

### Clinical consultation

Given a general lack of experience with treatment considerations specific to transgender youth, GSDP behavioral health clinicians have devoted efforts to building the capacity of practitioners in the Midwestern region. These efforts include the following:

(1) Peer Consultation: Following a patient's initiation of care in GSDP, our behavioral health clinicians attempt to establish phone-based peer consultation with a patient's primary therapist (when one exists). This consultation consists of provider education regarding gender development, transgender identity, GD course, social and medical aspects of gender transition, and consultation on conducting an assessment of readiness for youth pursing medical/surgical interventions.
(2) Gender-Affirming Clinicians' Collaborative: Established in fall 2015, this free, quarterly consultation group comprises Midwest regional behavioral health clinicians with varying degrees of experience conducting assessment and treatment with gender-diverse youth. Each confidential 90-minute call—organized and moderated by GSDP—consists of two prescheduled case discussions in which consultation is sought following a brief presentation of de-identified case material.
(3) Trainings: Program clinicians lead paid and *pro bono* trainings on topics such as transgender cultural competency, pediatric GD, and multidisciplinary approaches to transgender healthcare in community health, school, and legal settings.

### Gender euphoria therapy group

In 2016, program psychologists developed a weekly therapy group for transgender and gender-questioning adolescents. The group is guided by a social cognitive model with topics germane to this age group, including gender identity exploration, expression, and disclosure, family relationships, adaptive coping, social and medical transition, and dating and relationships.

### Gender-affirming surgical consultation

Some, but not all, transgender individuals desire gender-affirming surgical interventions. WPATH guidelines^8^ recommend that genital surgery be deferred until youth reach the legal age of majority, with more flexibility for minors pursuing gender-affirming chest surgery with parental consent. Many transmasculine patients presenting for care after pubertal maturity express a strong desire for chest surgery to reduce GD and improve body satisfaction. In 2015, our program partnered with a surgeon from Lurie Children's Division of Plastic and Reconstructive Surgery who has expertise in developmental breast anomalies to offer gender-affirming chest surgery and breast construction. To date, 24 patients have been seen for consultation and 7 have completed gender-affirming chest surgery. Program clinicians also make referrals to a local surgeon specializing in the full range of gender-affirming surgical procedures^15^ for patients ages 18 and older.

### Fertility preservation

Many gender-affirming medical and surgical interventions available to transgender youth affect future fertility.^16^ Clinical practice guidelines^6,8^ highlight the importance of discussing potential loss of fertility as a side effect of treatment. In response to patients' and families' concerns about potential loss of fertility, GSDP partnered with the Lurie Children's Fertility Preservation Team and Northwestern University's Oncofertility Consortium^*^ to routinely offer consultation and, if interested, fertility preservation services to patients before undergoing fertility compromising treatments. To date, nine patients have consulted with the Fertility Preservation Team nurse navigator, with two birth-assigned male patients pursuing sperm cryopreservation and one birth-assigned female patient pursuing oocyte cryopreservation.

## Adjunctive Programming

### Teen and parent support group

Many gender-diverse youth and their families are socially isolated and lack supportive programming. To meet this need, the GSDP PM facilitates a monthly teen and parent night for gender-diverse youth ages 13+ and their parents. Parents engage in peer-based support, and guest speakers often join parents to present topics that are germane to supporting gender-diverse youth. Teens are provided with structured programming each month, such as sexual health 101 and panel discussions with transgender adults. Established in 2013, the group has grown from an average of 10 participants to more than 40 each month.

### BeHeard: Voice and communication therapy

Many gender-diverse youth report dissatisfaction with voice quality. The WPATH Standards of Care^8^ highlight the clinical value of voice and communication therapy in alleviating GD and improving comfort with one's affirmed gender identity. In spring 2015, GSDP began offering an 8-week, group-based voice and communication therapy service for youth ages 12+. Formed in partnership with speech therapy faculty at Northwestern University's Center for Audiology, Speech, Language, and Learning, BeHeard's content focuses on improving aspects of verbal (e.g., pitch, resonance, intonation) and nonverbal communication (e.g., facial expression, posture, gestures).^8^

### BeStrong: Physical wellness group

Transgender youth are more likely to experience body-related dissatisfaction compared with cisgender peers.^17^ Many transgender youth find exercising in public uncomfortable, and this discomfort may be compounded by lack of access to trans-friendly locker facilities. To address these issues, in summer 2016, GSDP will offer a physical wellness group to gender-diverse youth ages 14+. This weekly, 10-session physical wellness program focuses on developing personal wellness goals, increasing knowledge of cardiovascular and weight training techniques, improving nutrition practices, and facilitating social support. Each session, run by a personal trainer certified by the National Academy of Sports Medicine (NASM) and who has received cultural competence training from GSDP team members, will consist of 45 min of cardiovascular/weight training and 15 min of nutritional education.

## Education and Community Outreach

Education and community outreach is an important component of GSDP as transgender youth continue to lack support and face discrimination in many communities. School and district personnel are often insufficiently trained to adequately address the needs of gender-diverse students, and, as a result, many school environments are not fully inclusive for all young people, regardless of gender identity or expression. To address this issue, our PM has designed professional development training sessions aimed at improving understanding of these complex issues and enabling educators to meet the needs of gender-diverse youth. School personnel are provided with practical solutions to interrupt and prevent gender discrimination, and, when requested, our PM provides ongoing postsession consultation.

Our PM also supports youth and families engaged in care with GSDP by directly advocating on their behalf both within schools and community organizations. Frequent issues include bathroom and locker room access, overnight trips, and use of a student's preferred name and gender pronoun. These issues are generally resolved by providing administrators with a better understanding of the rights and needs of gender-diverse and transgender youth and offering specific solutions. To support these efforts, the PM worked in collaboration with Illinois Safe Schools Alliance to draft a model school policy and administrative procedure. Released in April 2016, this document provides administrators with best practices for establishing inclusive school environments that affirm gender-diverse students.

Our PM also has been active in collaborating with local and national organizations in developing policies supporting gender inclusivity. Due in large part to our PM's efforts, Lurie Children's became the first children's hospital in the United States to release a policy statement in support of transgender youth in March 2016. To date, our PM has worked with over 120 schools and organizations. Of note, GSDP did not initially anticipate the growth potential of the education and community outreach arm of our program; however, given the success of our PM in directly impacting the health and well-being of our patients through community engagement, our team would recommend burgeoning gender programs consider dedicating staff to these efforts.

## Conclusion

Our team intentionally developed a model of care with flexible points of entry to meet the unique needs of gender-diverse and transgender youth and their families. While GSDP was initially established to address the growing demand for medical and mental healthcare for gender-diverse youth, our team quickly learned from our patients and families that their needs expanded beyond medical and mental health services. In particular, the education and community outreach arm of our program has grown exponentially in a way that was not initially anticipated and plays an integral role in positively impacting the health and well-being of our patients and families. Each unique aspect of our programming evolved in response to patient needs and taking a comprehensive approach to patient care.

## Notes

^*^ The Oncofertility Consortium. 2015. www.oncofertility.northwestern.edu (accessed March 1, 2016).

## Abbreviations Used

APN: Advanced Practice Nurse
GSDP: Gender and Sex Development Program
GDC: gender development clinic
GD: gender dysphoria
GnRHa: Gonadotropin-releasing hormone analogues
PM: program manager
WPATH: World Professional Association of Transgender Health
